# Frequency of Primary Headache Syndromes in Patients with a Major Depressive Disorder

**DOI:** 10.7759/cureus.2747

**Published:** 2018-06-05

**Authors:** Ather Muneer, Ahsen Farooq, Junaid H Farooq, Muhammad Siddique Qurashi, Immad A Kiani, Javeria S Farooq

**Affiliations:** 1 Department of Psychiatry, Islamic International Medical College (riphah International University), Rawalpindi, PAK; 2 Department of Radiology, Islamic International Medical College (riphah International University), Rawalpindi, PAK; 3 Internal Medicine, Charleston Area Medical Center / West Virginia University, Charleston, USA; 4 Internal Medicine, Shifa International Hospital, Islamabad, Pakistan, Islamabad, PAK

**Keywords:** primary headache, migraine, tension-type headache, major depressive disorder

## Abstract

Purpose

The primary objective of this study was to assess the overall frequency of primary headaches in subjects with a moderate to severe major depressive disorder. A further objective was to determine the frequency of primary headache sub-types in this population.

Materials and methods

This descriptive, cross-sectional study was conducted at the outpatients’ clinic of the department of psychiatry, Pakistan Railways Teaching Hospital, an affiliate of Islamic International Medical College, Rawalpindi. The duration of the study was from December 2016 to May 2017. One hundred and ten consecutive patients with a major depressive disorder (MDD) were assessed for a primary headache, according to the diagnostic criteria of International Classification of Headache Disorders second edition (ICHD-2). MDD patients with moderate to severe depression according to the diagnostic and statistical manual of mental disorders (DSM-5) were enrolled in the study. A semi-structured proforma was designed to gather information on sociodemographic variables. The data was analyzed by utilizing Statistical Package for Social Sciences, version 22 (IBM Corp., Armonk, NY, US).

Results

Of the 110 MDD patients enrolled, a primary headache was present in 45 (40.90%) cases. Additionally, five of these patients had a migraine with aura (11.11%), 12 had a migraine without aura (26.66%), and 28 had a tension-type headache (62.22%). In the females, migraines with or without aura was frequent (35.29%), while a tension-type headache was more common in males (72.72%).

Conclusion

More than one-third of the sample had a primary headache syndrome, which shows a high comorbidity between a migraine and its variants and MDD. There is a need to undertake further studies with larger samples to elucidate this relationship.

## Introduction

As defined in the medical literature, headache or cephalalgia is the experiencing of pain sensation anywhere in the region of the head. With respect to etiology, primary headache is considered as not having an underlying organic origin, while a secondary headache is due to a causal pathological lesion [1]. Most importantly, primary headache syndromes comprise a migraine, with or without aura, a tension-type headache (TTH), a cluster headache, and a combined tension-type/migraine variant [2]. Headache disorders are a source of great morbidity and have been aptly ranked amongst the 10 most disabling conditions in the world by the World Health Organization [3]. Primary headache disorders are rampant globally and have an estimated prevalence of 46% in the adult population worldwide. In this respect, TTH and migraine are the common most, affecting approximately 42% and 11% of the adult population, respectively [4]. Furthermore, epidemiological studies suggest that primary headache disorders have a lifetime prevalence of 90%. In spite of the aforementioned facts, these disorders are on the whole neglected and uncared for ailments, placing a huge burden on the patients and their families [5].

A major depressive disorder (MDD) is an affective disorder, exemplified by subjectively low mood, lack of interest and pleasure in day-to-day activities, reduced energy, easy fatigability, feelings of guilt, and disturbed sleep and appetite, leading to an impairment in psychosocial functioning. The lifetime prevalence of MDD is about 20% and point prevalence is 2%-5%, having a greater frequency in females as compared to males [6]. Data from epidemiological and clinical studies have demonstrated a link between primary headache syndromes and MDD. In this regard, TTH and migraine have been suggested as having a particularly close connection with major depression, and emerging evidence indicates that these conditions have common neurobiological underpinnings [7]. Recent research has shown that generalized anxiety disorder (GAD) and MDD are highly comorbid with migraines, with prevalence rates as high as 55% [8]. Although firm evidence is lacking, the association between primary headache syndromes and affective disorders may be bi-directional, meaning thereby that increased severity of one leads to another and vice versa. While space limitation does not allow the details of the pathophysiologic mechanisms, it is clear that primary headache syndromes, especially migraine and mood disorders are, in essence, progressive conditions and likely share common pathogenic pathways [9]. Specifically, with migraines, a revealing study from South Asia showed that a majority of the participants were female, had a migraine without aura (> 80%), and only 22% did not have a concurrent psychiatric diagnosis. In this study, the most common psychiatric diagnoses were GAD, mixed anxiety, depressive disorder, and depressive episodes [10]. It must be acknowledged that a majority of the data come from a small number of high-income countries so that the epidemiological characteristics and disease burden of primary headache disorders are mostly unclear in the developing world.

With this preamble, the purpose of the current study was to estimate the frequency of primary headache disorders in patients presenting with MDD in a psychiatry outpatient clinic in order to determine the prevalence of these conditions in the said population. A further objective was to determine the correlates of this relationship to clarify any risk factors linked to this association.

## Materials and methods

This descriptive, cross-sectional study was conducted at the psychiatry outpatient department of the Pakistan Railways Teaching Hospital, Rawalpindi, from December 1, 2016, to May 15, 2017, and comprised 110 patients with a major depressive disorder. After obtaining ethical approval from the institutional review board, patients were enrolled using non-probability convenience sampling. Patients with MDD in the 18-60 years age range and having moderate to severe disorder were included. Patients having a history of chronic medical conditions or substance abuse were excluded. Similarly, patients having psychotic features were also excluded. Informed consent was obtained from patients after telling them in simple and understandable language about the purpose of the study, assuring them of confidentiality and recognizing their right to withdraw the consent at any time, even without mentioning any reason for that. The diagnosis was based on a comprehensive clinical interview and a relevant clinical examination using the diagnostic criteria of International Classification of Headache Disorders second edition (ICHD-2) for a primary type of headache disorders and Diagnostic and Statistical Manual – 5th Edition for MDD. Later, available magnetic resonance imaging (MRI) scans of all the patients were read by the consultant radiologist to rule out any organic cause of these headaches. A semi-structured proforma was used to gather demographic details of the patients. The data were analyzed with SPSS version 22. The frequency and percentage were calculated for qualitative variables like gender, headache type, marital status, educational status, occupational status, etc. Mean and standard deviation (SD) were calculated for quantitative variables, for example, age.

## Results

Of the 110 participants, 41 (37.27%) were males and 69 (62.72%) were females. The mean age of the subjects was 36 ± 14.0 years (range 22 to 50 years). Furthermore, 84 (76.36%) participants were married and the rest were single. Twenty-five subjects (22.72%) were uneducated and, of the females, the majority (72.46%) were housewives. Among all the patients, 69 (62.72%) were self-referrals. In addition, 76 (69.09%) patients had moderate MDD and 30.90% had severe MDD (Table 1). A primary headache was present in 45 cases (40.90%). Additionally, five of these patients had a migraine with aura (11.11%), 12 had a migraine without aura (26.66%), and 28 had a tension-type headache (62.22%). In the females, migraine with or without aura was frequent (35.29%), while a tension-type headache was more common in males (72.72%). The gender-wise distribution of primary headache disorders in study subjects is given in Table 2. In the participants with a headache, 20 females(58.82%) were severely depressed and 41.17% were moderately depressed. In the males, 3 (27.27%) were severely depressed and the rest (72.72%) had moderate MDD. In both sexes, there was no significant association of primary headache disorders with the severity of depression.

**Table 1:** Demographic Characteristics of Participants

GENDER	N (%)
Male	41 (37.27)
Female	69 (62.72)
MARITAL STATUS	
Single	26 (23.63)
Married	84 (76.36)
EDUCATION	
Uneducated	25 (22.72)
Middle	15 (13.63)
Matric	48 (43.63)
Intermediate	15 (13.63)
Graduate	7 (6.36)
OCCUPATIONAL STATUS	
Unemployed	12 (10.90)
Student	8 (7.27)
Housewife	50 (45.45)
Own business	29 (26.36)
Professional	11 (10.0)
MODE OF REFERRAL	
Self-referral	69 (62.72)
Family-referral	27 (24.54)
Other (friend, etc.)	14 (12.72)
MDD SEVERITY	
Moderate MDD	76 (69.09)
Severe MDD	34 (30.90)

**Table 2:** Gender-wise Frequency of Primary Headache Syndromes in Depressed Patients

PRIMARY HEADACHE SYNDROME	MALE (n = 41)	FEMALE (n = 69)
No headache	30 (73.17%)	35 (50.72%)
Any primary headache	11 (26.82%)	34 (49.27%)
Migraine with aura	1 (9.09%)	4 (11.76%)
Migraine without aura	2 (18.18%)	8 (23.52%)
Tension-type headache	8 (72.72%)	22 (64.70%)

## Discussion

This study is one of the few where the frequency of a primary headache was determined in an outpatient sample with major depression without psychotic features. It shows a high prevalence of headache disorders in moderate to severely depressed patients. A primary headache was present in 40.90% of the cases, and this rate is somewhat similar to a recently published study conducted in a tertiary care hospital in Karachi, in which 37.16% of the depressed subjects had a primary headache [11].

A cross-sectional study conducted in Italian secondary and tertiary care centers assessed patients with primary headache syndromes, including migraines without aura, tension-type headaches, and combined TTH and migraine with a validated scale, i.e., Mini International Neuropsychiatric Instrument (MINI), and found that major depression was present in about 1/3^rd^ of the sample [12]. Furthermore, it revealed that patients with combined migraine and TTH had a higher prevalence of MDD, which could be due to shared environmental and genetic risk factors and pathophysiologic mechanisms. An Asian study attempted to determine the correlation between anxiety and depression and headache characteristics in migraineurs. It utilized Beck Depression Inventory and Hospital Anxiety and Depression Scale in outpatients having migraine with or without aura and found out that a higher migraine frequency was significantly associated with greater symptom scores of anxiety and depression [13].

Yet another study conducted in a European country systematically examined several disease characteristics in migraineurs, for example, pain intensity, attack frequency, attack duration, presence or absence of aura, and disease duration. No specific correlation with anxiety/depression was found for any of the above factors, although moderate levels of depression and anxiety were more frequent in the migraine population generally. Since this was a small study, the authors concluded that larger samples were required in future studies to assess the link between specific headache characteristics and mood and other psychiatric disturbances [14]. In our study too, we could not find any association with the severity of depression and primary headache syndromes, and it can be implied that while there is a connection between mood disorders and headache, the specific features of this connection require further investigation. A large study conducted in the Netherlands studied more than 2500 patients with a migraine and found that 45% suffered from lifetime depression. In that study, higher depression frequency was associated with a greater number of migraine attacks, female gender, high body mass index, being single, and cigarette smoking. In addition, those with comorbid depression and migraine had allodynia or greater sensitization to pain [15].

A migraine is often chronic, which implies that a headache occurs for 15 or more days per month for longer than three months. In this population, a study showed that more than 85% of the patients had depression which was of moderate to severe intensity in 58.7% of the cases [16]. In our study, we enrolled patients having moderate and severe MDD and were able to show that >40% suffered from migraines with or without aura, TTH, or combined TTH and migraine syndrome. These facts point out that the comorbidity between a headache and depression is bidirectional, a very large number of subjects suffering from a chronic headache develop depression, and vice versa. Lastly, the American Migraine Prevalence and Prevention study followed 24,000 subjects with a severe headache prospectively for a number of years. After controlling for sociodemographic variables and headache characteristics, it was demonstrated that conversion from an episodic to a chronic migraine was significantly associated with depression and that increased severity of depression was a risk factor in this regard [17]. This means that the presence of comorbid depression is a risk factor for the progression of a migraine, and clinicians must be alert to this occurrence in order to effectively treat their patients.

## Conclusions

This study is unique in the sense that very few investigators have attempted to determine the frequency of primary headache syndromes in patients with major depression. While a literature search revealed that several well-designed studies investigated the occurrence of psychiatric disorders in populations suffering from various types of primary headaches, our study checked for this association in a depressed sample. However, in line with the reported work, we found that headache was frequently comorbid with depression. This finding has important connotations for clinicians since they should be aware of this association. With respect to neurologists, internists, and general practitioners, these professionals must be on the lookout for depressive disorders in their patients with primary headache syndromes, be able to diagnose MDD, and either treat this comorbidity themselves or make referrals to psychiatrists. Without appropriate therapeutic measures, migraines and comorbid depression are likely to progress and become chronic and very disabling.
